# Screening for *Trypanosoma cruzi* infection (Chagas disease) in the Latin American population living with HIV in London

**DOI:** 10.1111/hiv.70201

**Published:** 2026-01-29

**Authors:** Natalie Elkheir, Shivani Singh, Birgit Barbini, Lucy Campbell, Alice Sharp, Jacqueline O'Connell, Louise Terry, Margherita Bracchi, Javier Rubio, Nicolo Girometti, Mohammed Hassan, Hannah Davies, Frank A. Post, Laura Nabarro, Debbie Nolder, Geraldine O'Hara, Julie Fox, Peter L. Chiodini, David A. J. Moore

**Affiliations:** ^1^ Clinical Research Department London School of Hygiene & Tropical Medicine London UK; ^2^ Hospital for Tropical Diseases University College London Hospitals London UK; ^3^ UK Chagas Hub, https://www.uclh.nhs.uk/uk‐chagas‐hub; ^4^ Harrison Wing, Guy's and St Thomas' NHS Trust London UK; ^5^ Caldecot Centre, King's College Hospital NHS Foundation Trust London UK; ^6^ Chelsea & Westminster Hospital NHS Foundation Trust London UK; ^7^ Department of Clinical Parasitology Health Services Laboratories London UK; ^8^ Diagnostic Parasitology Laboratory London School of Hygiene & Tropical Medicine London UK; ^9^ Department of Infection Guy's and St Thomas' NHS Trust London UK

**Keywords:** Chagas disease, co‐infection, epidemiology, HIV, Latin American, migrant health, prevalence, screening, *Trypanosoma cruzi*

## Abstract

**Background:**

There are an estimated 2482 people born in Latin American countries receiving care for HIV in the United Kingdom. Although national guidance recommends screening for *Trypanosoma cruzi* infection (Chagas disease) in this population, there is no formal screening programme. The aim of this study was to investigate the sero‐epidemiology of *T. cruzi* in Latin American people living with HIV in London, with a view to informing screening strategies.

**Methods:**

This was a cross‐sectional study, using serological screening for *T. cruzi* and questionnaires. Adults receiving HIV care in three London hospital outpatient clinics and who were born in one of the 21 Chagas‐endemic countries of South America, Central America or Mexico were identified by searching electronic medical records for country of birth codes. Eligible participants were invited to undergo *T. cruzi* screening (a single recombinant immunoglobulin G (IgG)‐enzyme‐linked immunosorbent assay (ELISA)) during routine HIV outpatient appointments. Positive screening tests were confirmed with an immunofluorescence antibody test, with discordant results resolved by an adjudicating immunoblot.

**Results:**

A total of 351 participants were recruited between June 2023 and March 2025, including 181 (52%) who were born in Brazil, 73 (21%) in Colombia, 19 (5%) in Ecuador, 16 (5%) in Argentina, 15 (4%) in Venezuela and 12 (3%) in Bolivia. Uptake of serological screening was high (97% when offered face to face). Four participants (1%) had a positive screening test, of whom one had confirmed *T. cruzi* infection (seroprevalence 0.3%, 95% CI 0.01–1.6%), and three were deemed false positive screening tests.

**Conclusions:**

We report a low seroprevalence of *T. cruzi* infection in Latin American migrants in London living with HIV. Since the clinical consequences of untreated *T. cruzi* infection can be fatal, screening uptake is high, and individuals in non‐endemic countries generally only need to be screened once, we recommend testing all Latin American people with HIV, especially women of reproductive age, those from high‐prevalence countries and those with poor immune‐virological control.

## INTRODUCTION

Chagas disease is a neglected tropical disease endemic to continental Latin America caused by the protozoan parasite *Trypanosoma cruzi*. It is acquired from triatomine insect vectors, vertical transmission and, in the absence of screening, through blood transfusion and organ transplantation [1]. Due to globalization and migration, Chagas disease is increasingly recognized as a public health problem among migrants in Europe and the United States [2, 3]. An estimated seven million people worldwide have *T. cruzi* infection and 12 000 people die each year from clinical manifestations of Chagas disease [4]. The consequences of this chronic infection include progression, over 10–40 years, to cardiac or gastrointestinal end‐organ disease in approximately one third of infected individuals [5]. Cardiac complications include conduction defects, dysrhythmias (a potential cause of sudden cardiac death) and dilated cardiomyopathy [6]. Gastrointestinal complications include oesophageal and colonic dysmotility and dilatation [5].

While classic cardiac and gastrointestinal presentations of chronic Chagas disease occur in people living with HIV, they are additionally at risk of *T. cruzi* reactivation, a severe and often fatal form of *T. cruzi*‐mediated disease [7]. *T. cruzi* reactivation typically occurs with low CD4 counts and poor virological control. In a recent systematic review of HIV/*T. cruzi* co‐infection, 86% of published cases of reactivations were reported in individuals with CD4 < 200 cells/mm^3^ and no cases of reactivation were published in individuals with HIV viral suppression [8]. The early detection of *T. cruzi* infection in people with HIV enables administration of trypanocidal therapy, which substantially reduces the risk of future reactivation disease [7, 8].

According to the Office for National Statistics (ONS) 2021 Census, England and Wales are home to over 280 000 people born in Chagas‐endemic countries (21 countries of Central America, South America and Mexico), approximately half living in London [9]. The largest groups are people born in Brazil, Colombia and Ecuador, who make up 37%, 18% and 11% of the London Latin American population, respectively [9]. There are an estimated 2482 people born in Latin American countries receiving care for HIV in the United Kingdom, 89% of whom are men, and 72% of whom receive HIV care in London [10]. Although British HIV Association (BHIVA) guidance recommends *T. cruzi* screening for ‘all HIV‐seropositive people with epidemiological risk factors for Chagas disease’, [11] there is no formal screening programme, nor consensus on how best this might be done, resulting in minimal screening of the at‐risk population.

The aim of this study was to investigate the sero‐epidemiology of *T. cruzi* in Latin American migrants living with HIV in London and to inform the development of potential screening strategies.

## METHODS

### Study design

This was a sero‐epidemiological study, utilizing serological screening tests (for *Trypanosoma cruzi* infection) and questionnaires.

### Study population

Adults born in the 21 endemic countries* of Central America, South America and Mexico that were receiving care for HIV in London were invited for serological screening and questionnaire completion. Individuals declining to participate or lacking capacity to give consent were excluded.

*Argentina, Belize, Bolivia (Plurinational State of), Brazil, Chile, Colombia, Costa Rica, Ecuador, El Salvador, French Guiana, Guatemala, Guyana, Honduras, Mexico, Nicaragua, Panama, Paraguay, Peru, Suriname, Uruguay and Venezuela (Bolivarian Republic of).

### Setting

This study recruited participants from outpatient HIV clinic appointments at three secondary care trusts in London.

### Recruitment

Eligible participants were identified through country of birth data captured in the initial HIV clinic assessment (routinely captured and coded), via a search of the electronic medical records. Eligible patients were approached during routine clinic appointments or sent an automated text message with an online link to the study information and an option to self‐complete the questionnaire with a blood draw to follow at the next routine clinic appointment.

### Sample size calculation

No comparable screening studies have been done in HIV clinics with similar demographics to this population of people living with HIV. The sample size calculation was therefore based on the total pooled prevalence from a meta‐analysis of *T. cruzi* prevalence of Latin American migrants living in Europe, which was 4.2% (95% CI: 2.2–6.7%) [12]. For this expected prevalence, with a precision of 2.1% (half the prevalence) and confidence of 95%, the sample size required was 351.

### Data collection methods

#### Key participant information

Eligible individuals were asked (in a questionnaire conducted in Spanish, Portuguese or English) for their contact details, key demographics, migration history, details on previous testing for Chagas disease and family history of Chagas disease.

#### Serological screening

Participants were serologically screened with an IgG recombinant‐enzyme‐linked immunosorbent assay (ELISA), the Chagatest ELISA recombinant v.4.0 according to the manufacturer's instructions [13] (performed at the parasitology laboratory of the Hospital for Tropical Diseases, HTD), which has a reported sensitivity of 99% and specificity of 98%.

A positive ELISA was followed by an in‐house immunofluorescence antibody test (IFAT) at HTD, and qualitative PCR at the Diagnostic Parasitology Laboratory at the London School of Hygiene & Tropical Medicine. Discordant serology results were resolved by the result of an adjudicating immunoblot (the LDBIO Diagnostics Chagas Western Blot IgG). In accordance with the World Health Organization's diagnostic guidance at the time of the study, two positive serological tests were required to confirm the diagnosis of Chagas disease [14].

#### Chagas disease clinic follow‐up

Individuals with a positive screening test were referred to the Chagas clinic at HTD as part of routine National Health Service (NHS) care. Routine investigations (in the management of Chagas disease) for all seropositive patients were offered (12‐lead ECG, ambulatory ECG monitoring, chest x‐ray & echocardiogram).

### Primary outcome

Prevalence of *T. cruzi* seropositivity (defined as ELISA positive, plus IFAT or immunoblot positive) was the primary outcome.

### Data analysis

Serological screening resulted in binary classification of the primary outcome (seropositive/seronegative). Confidence intervals around the seroprevalence estimate were calculated using the Clopper‐Pearson exact method [15] using the ci proportion command in STATA.

Continuous data for all participants were presented as the mean and standard deviation (if normally distributed) or median and interquartile range (if not), and categorical data as counts and frequencies. All analyses were performed using STATA version 18 (StataCorp, College Station, TX, USA).

### Ethical approval

Ethical approval for this study was granted from the London School of Hygiene & Tropical Medicine's Ethics Committee (ref: 2022‐KEP‐904) and the national Health Research Authority (IRAS ID: 324455).

## RESULTS

### Identification of eligible participants for screening

A search of electronic medical records identified 1228 eligible participants (based on country of birth codes): 266 at site one, 184 at site two and 778 at site three.

### Uptake of testing

A total of 351 participants were recruited into the study between June 2023 and March 2025 (161 in site one, 87 in site two and 103 in site three) (Table 1). At sites one and two, where participants were systematically identified in advance of routine HIV appointments and approached face to face in these appointments by the research team, uptake of screening was 97% overall (98% in site one and 97% in site two). At site three, 13 patients responded to a text message sent with a link to information (out of 110 total texted, uptake 12%). HIV clinicians at site three opportunistically recruited 90 additional eligible participants in clinic appointments (and no one refused participation when approached this way).

**TABLE 1:** Characteristics of 351 participants with HIV screened for *Trypanosoma cruzi* infection in London, 2023–2025.

Participants recruited by site, *n* (%)		
Site one		161 (45%)
Site two		87 (25%)
Site three		103 (29%)
Age at screening, mean (SD)		45 (11)
Gender, *n* (%)	Male	327 (93%)
	Female	21 (6%)
	Non‐binary	3 (1%)
Country of birth, *n* (%)	Brazil	181 (52%)
	Colombia	73 (21%)
	Ecuador	19 (5%)
	Argentina	16 (5%)
	Venezuela	15 (4%)
	Bolivia	12 (3%)
	Mexico	8 (2%)
	Peru	8 (2%)
	Other country	19 (5%)
**Self‐reported (questionnaire) risk factors for Chagas disease**		
Years since last lived in Chagas‐endemic^a^ country	Mean (SD)	20 (11)
	Median (IQR)	20 (12–27)
	Range	2 months–51 years
Tested for Chagas disease before, *n* (%)	Yes	23 (7%)
	No	253 (72%)
	Don't know	54 (15%)
If tested before, result, *n* (%)	Positive	1 (4%)
	Negative	21 (91%)
	Don't know	1 (4%)
If positive, antiparasitic treatment received, *n* (%)	Yes	1 (100%)
First‐degree relative with Chagas disease	Yes	12 (3%)
	No	263 (75%)
	Don't know	55 (16%)
	Did not answer	21 (6%)

*Note*: Gender is self‐reported as per questionnaire response. Male includes trans male. Female includes trans female. Countries of birth with fewer than five participants each have been combined in the ‘other country’ category to prevent identification.

^a^ 21 countries of South America, Central America and Mexico: Argentina, Belize, Bolivia (Plurinational State of), Brazil, Chile, Colombia, Costa Rica, Ecuador, El Salvador, French Guiana, Guatemala, Guyana, Honduras, Mexico, Nicaragua, Panama, Paraguay, Peru, Suriname, Uruguay and Venezuela (Bolivarian Republic of).

### Characteristics of participants recruited to screening

Most participants were male (*n* = 327, 93%) with a mean age of 45 (SD 11) years. Over half the participants were born in Brazil (*n* = 181, 52%), with 21% born in Colombia, and fewer than 20 participants born in each of the other Latin American countries represented (Table 1). No participants were known to have a detectable viral load at the time of screening.

**FIGURE 1:**
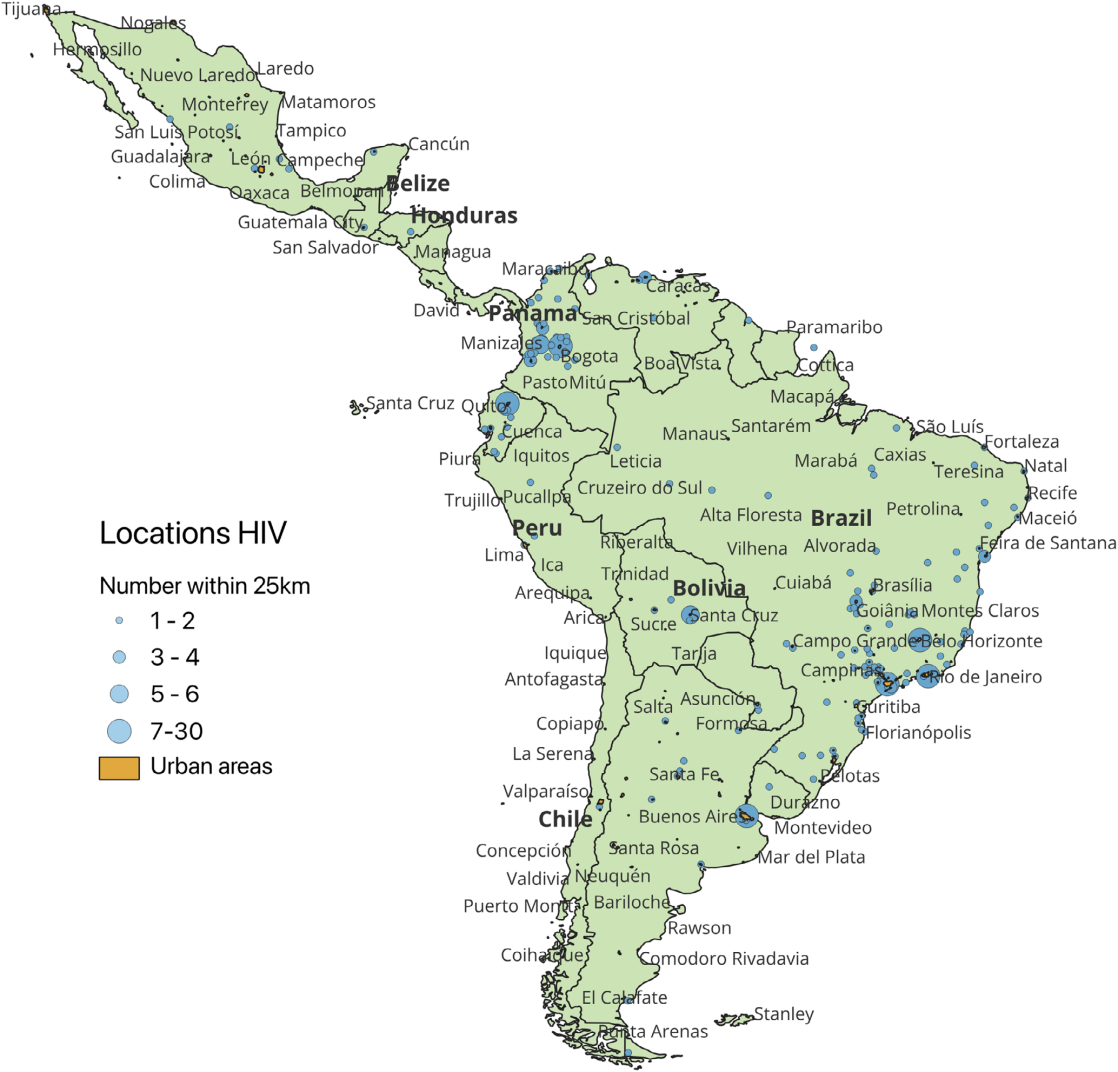
Locations in Latin America where participants recruited to *T. cruzi* screening from HIV clinics had lived (self‐reported in questionnaire). Participants were asked to select up to three locations on a digital interactive world map (coordinates) where they had lived in Mexico, Central America or South America. These self‐reported coordinates are displayed on a map generated in QGIS software (version 3.34.13‐Prizren). To ease visualization of data, coordinates within 25 km of each other are clustered as per the key—larger plots represent more participants having selected that location. Urban areas (from Natural Earth Data) are represented as an overlay to the map in orange. These maps are freely and openly distributable under GNU General Public License.

The range of time since participants last lived in an endemic country was large (between 2 months and 51 years), with a mean time of 20 years (Table 1). Twenty‐three participants (7%) reported previously having been tested for Chagas disease, with one participant reporting a positive test and receipt of antiparasitic treatment. Twelve participants (3%) reported having a first‐degree relative with Chagas disease. Most participants reported having lived only in major urban cities (Figure 1).

### Follow‐up of screening results

Of 351 participants serologically screened for *T. cruzi*, four had a reactive ELISA and 347 were negative. Three out of the four participants had weakly reactive ELISAs and were deemed false positive results (as the subsequent IFAT and immunoblot were negative). In the fourth participant, born in Bolivia, the diagnosis of Chagas disease was confirmed (ELISA and IFAT positive); however, this individual was lost to follow‐up. One additional participant (whose ELISA was negative) reported a previous diagnosis of Chagas disease and antiparasitic treatment years previously in Bolivia, and so this individual was linked into specialist care for long‐term follow‐up.

### Screening test characteristics

One case of *T. cruzi* infection was confirmed out of 351 participants screened, an overall seroprevalence of 0.3% (95% CI 0.01–1.6%). The positive predictive value of the recombinant ELISA screening test was 25% (one serologically confirmed case out of four participants with reactive ELISA).

### Linkage to care

Of the five participants requiring confirmatory testing for *T. cruzi*, three (60%) were successfully engaged in follow‐up and two (40%) were lost to follow‐up.

## DISCUSSION

This sero‐epidemiological study, the largest of its kind conducted in the United Kingdom, found a low seroprevalence of *Trypanosoma cruzi* (0.3%) among Latin American individuals living with HIV. Uptake of serological screening was very high, demonstrating the feasibility of implementing systematic screening in large secondary care clinic populations. The study also underscored the importance of confirmatory testing using a second, distinct serological assay, as three of the four initial positive results were ultimately identified as false positives.

The low *T. cruzi*/HIV co‐infection prevalence identified in this study aligns with findings from similar studies in non‐Chagas‐endemic settings. The only other UK study assessing *T. cruzi*/HIV co‐infection prevalence, a smaller cross‐sectional study at a single London clinic, screened 86 out of 231 identified participants using the same recombinant‐ELISA and found no seropositive cases [16]. In a study from Italy, in which 389 Latin American migrants with HIV were screened for *T. cruzi*, the detected seroprevalence (with two different parallel ELISAs positive) was 0.51% [17]. The positive predictive value of a single recombinant‐ELISA was 15%, similar to our finding. Notably, the use of parallel screening in that study did not improve sensitivity, as none of the discordant results were confirmed on a third, discriminatory immunoblot. As is the case in this study, the high rate of false positive screening ELISAs may be due to cross‐reactivity.

Higher *T. cruzi* seroprevalence, ranging from 1.9% to 11%, has been reported among people with HIV in Spain [18, 19, 20], likely reflecting differences in migrant populations, particularly higher proportions of individuals from endemic rural areas of Latin America. In contrast, the very low prevalence observed in our study likely reflects the urban origin of the Latin American people with HIV in London, most of whom come from low‐prevalence regions, and does not justify routine serological screening in this context. However, since individuals in non‐Chagas‐endemic countries only need to be tested once (to cover previous exposure risk), and given the high acceptability and feasibility of screening, offering testing to all Latin American people with HIV is recommended. This should be incorporated, for Latin American individuals, into the routine multi‐disease screening offered to all patients with HIV. The success of any screening initiative will rely on a parallel retention in care strategy for any individuals who test positive for *T. cruzi*. Confirmed cases should also trigger family screening, potentially identifying additional people who would benefit from antiparasitic therapy and management of end‐organ disease. In settings where offering testing to all Latin American people with HIV for *T. cruzi* is not feasible, risk‐stratification based on epidemiological considerations such as country of birth, having lived in a rural area/adobe house, having seen the triatomine vector, etc. (as adopted in some Spanish centres [19]) is a practical alternative. Additionally, testing all individuals with low CD4 counts or poor virological control is essential due to the higher risk of reactivation. All women from Latin America of reproductive age (and pregnant women) should also be offered testing, due to the additional benefit of interrupting vertical *T. cruzi* transmission.

A key limitation of this study is that clinical variables (such as CD4 count or AIDS diagnosis) were not collected. Although screening uptake was very high, the possibility of sampling bias still exists and is a further limitation. The main strength of this study is the large sample size and the fact that the recruitment of participants took place at three sites in different parts of London, resulting in a sample representative of the wider London Latin American population living with HIV. The low seroprevalence of co‐infection estimated in this study is therefore a robust estimate. As well as estimating the seroprevalence of co‐infection, this study provided good insight into the acceptability of screening (high when offered face to face in clinic appointments) and reach of screening based on different approaches, allowing large clinic populations to be screened.

## CONCLUSION

We report a low seroprevalence of *T. cruzi* infection in Latin American migrants in London living with HIV. Since the clinical consequences of untreated *T. cruzi* infection can be fatal, screening uptake is high and individuals in non‐endemic countries generally only need to be screened once, we recommend testing all Latin American people with HIV, especially women of reproductive age, those from high‐prevalence countries and those with poor immune‐virological control.

## AUTHOR CONTRIBUTIONS

NE, SS, GoH and DAJM conceived of the study. NE, BB, LC, AS, JoC, MB, JR, NG and MH collected data. NE, HD, FAP, GoH, JF, PLC and DAJM analysed the data. DN and LN analysed the laboratory samples. NE drafted the manuscript. All co‐authors reviewed and approved the manuscript.

## FUNDING INFORMATION

NE was supported by a Clinical Research Training Fellowship from the Medical Research Council.

## CONFLICT OF INTEREST STATEMENT

No conflicts of interest declared.

## ETHICS STATEMENT

Ethical approval for this study was granted from the London School of Hygiene & Tropical Medicine's Ethics Committee (ref: 2022‐KEP‐904) and the national Health Research Authority (IRAS ID: 324455). All participants gave written informed consent.

## Data Availability

The data that support the findings of this study are available from the corresponding author upon reasonable request.
